# Urological referral of asymptomatic men in general practice in England

**DOI:** 10.1038/sj.bjc.6604291

**Published:** 2008-03-18

**Authors:** J Melia, P Coulson, D Coleman, S Moss

**Affiliations:** 1 Cancer Screening Evaluation Unit, Section of Epidemiology, Institute of Cancer Research, Sir Richard Doll Building, Cotswold Road, Sutton, Surrey SM2 5NG, UK

**Keywords:** primary health care, hospital referral, health education, prostate-specific antigen

## Abstract

The Prostate Cancer Risk Management Programme (PCRMP) launched in November 2002 provides guidelines for general practitioners (GPs) on age-specific prostate-specific antigen (PSA) cutoff levels in asymptomatic men. The impact of the PCRMP on GP referrals is unknown. This study investigates whether there was a change in the proportion of asymptomatic men with raised PSA levels (⩾3 ng ml^−1^) who were referred to urologists since the launch of the guidelines. Sixty-nine general practices in four areas of England and the main pathology laboratory in each area, which had participated in our previous research, were asked to provide data. Forty-eight practices (70%) provided retrospective data on urological referrals in men who had a PSA test taken in the periods 1 December 2001 to 31 May 2002 (pre-launch) and 1 December 2003 to 31 May 2004 (post-launch). Data on referrals were completed for 709 (79%) out of 898 and 1040 (90%) out of 1157 raised records pre- and post-launch, respectively. The percentage of men with raised PSA levels who were asymptomatic was similar in both time periods (19–20%) and the proportion referred to urologists according to the PCRMP guidelines did not increase significantly over time (24% pre-launch and 29% post-launch, *P*=0.42). The referral rate was lower than expected if the guidelines had been followed. The influence of the guidelines seems to have been low. At the time of data collection, 56% (112 out of 200) of GP partners reported that they were aware of receiving the PCRMP pack. To ensure future, effective implementation of guidelines requires evaluation.

The rate of prostate-specific antigen (PSA) testing for prostate cancer has been increasing in the United Kingdom since the early 1990s (Gavin *et al*, 2004; Melia *et al*, 2004; NHS Cancer Screening Programmes, 2004). Part of this increase will be due to testing for prostate cancer in asymptomatic men. In 28 areas of England and Wales, using data collected from 1 December 2001 to 31 May 2002, we had estimated the annual rate of PSA testing by general practitioners (GPs) in men aged 45–84 years with no previous diagnosis of prostate cancer to be 6%, with the rate of asymptomatic testing being 2% (Melia *et al*, 2004). In September 2002, the NHS Prostate Cancer Risk Management Programme (PCRMP) was launched (NHS Cancer Screening Programmes, 2008). All GPs and urologists in England were sent the PCRMP information pack, which contained materials to help men requesting a PSA test to make an informed choice about the benefits and potential harms of screening. The effect of the programme on knowledge and intention to be tested has been studied (Watson *et al*, 2006). The programme also provided guidelines on the age-specific PSA cutoff levels for urological referral in asymptomatic men: aged 50–59 years ⩾3 ng ml^−1^, 60–69 years ⩾4 ng ml^−1^ and 70 years or more >5 ng ml^−1^. The impact of the initiative on referral rates for men with a raised PSA is unknown.

The results presented in this paper compare the rates of urological referral between two 6-month periods before and after the introduction of the PCRMP, specifically in asymptomatic men. A randomised controlled trial could not be conducted because of the nationwide distribution of PCRMP packs. Data from our previous study in England (Melia *et al*, 2004) provided a baseline for the present study. Additional data on PSA testing were collected from a subset of the pathology laboratories and data on referrals from selected general practices. A full report on the study is available on the NHS Cancer Screening Programmes website.

## MATERIALS AND METHODS

### Study areas and selection of general practices

The pathology laboratory at the main hospital in each of the study areas Chichester, Sutton & Merton, Truro and York, which had provided timely data in the previous study (Melia *et al*, 2004), agreed to take part in the study. Data were collected on PSA requests from general practices in our previous study. The criteria for selecting the original group of practices were that they should routinely send all NHS PSA requests to the laboratory at the main hospital in their area, and that all GP partners should provide signed consent.

These practices were invited to participate in the present study. Each GP partner completed a one-page questionnaire on demographic data, membership of the Royal College of General Practitioners (MRCGP) and awareness of receiving the PCRMP pack.

### Data collection on PSA requests and referrals

Data on PSA requests from the pathology laboratories were collected for the period 1 December 2001 to 31 May 2004. The variables were specimen number, date of test, name and date of birth of man receiving test, NHS number and hospital number, name of GP and practice, and total PSA level (ng ml^−1^). Patient-based set of records was formed.

It was not feasible to ask the GPs to provide retrospective data on referrals for the full 2½ year period, so data were collected for one period pre-launch (1 December 2001 to 31 May 2002) and another period post-launch (1 December 2003 to 31 May 2004). The first time period was chosen because data had already been collected on reason for test (Melia *et al*, 2004). The second time period was chosen at the same time of year to avoid confounding from seasonal variation in rate of testing and to ensure that GPs would have had time to assess and actively use the PCRMP packs distributed in September 2002.

Referral data were collected on all men with a raised PSA value (⩾3 ng ml^−1^), all men with more than one test over the two time periods and a random 25% sample of men with a single low PSA value (<3 ng ml^−1^). Thus, data collection concentrated mostly on records with PSA levels above the minimum PCRMP age-specific level of 3 ng ml^−1^ to reduce the number of patient records to be searched. A pro forma was prepared for each man, listing his PSA tests (Appendix A). The form included definitions of the reasons for tests as used in the previous study (Melia *et al*, 2004). There were four categories: men with no previous diagnosis of prostate cancer who were asymptomatic, symptomatic or being re-tested, and men already diagnosed with prostate cancer. Data on urological referrals included the date of referral and hospital name. Reasons for non-referral or for not being able to complete a pro forma were reported. Each general practice was paid a flat rate per patient record checked.

It was not feasible to ask retrospectively whether the PCRMP pack had been used during patient consultations in the post-launch period because this was not recorded in patient notes.

A questionnaire on local initiatives was sent to the consultant urologists, chairmen of the Primary Care Trusts and Directors of Public Health. However, no initiatives were identified up to the end of the post-launch study period.

### Sample size

The sample size was selected to give 80% power to detect, at the 5% level of significance, a difference of 70 *vs* 83% in the proportions referred before and after the distribution of leaflets. Data collection resulted in a larger population being studied than originally planned with a larger number of PSA tests but a lower rate of urological referral (overall <30% in both study periods). The larger population resulted in the study being powered to detect changes from 20 to 40% in the proportion of men referred.

### Analysis

Poisson regression analyses were used to study the relation between GP partners’ awareness of receiving the PCRMP pack and GP characteristics, and the distribution of the proportion of GP partners per practice aware of the PCRMP packs has been studied.

Analyses were conducted on the first PSA test recorded for each man in each of the pre- and post-launch periods. The definition of ‘first recorded test’ is restricted to PSA tests reported within a strict time period and does not refer to the first PSA test ever taken for an individual. The men were aged 45–84 years at time of their first test, had a PSA test within either of the two time periods pre- and post-launch, and were not reported dead or no longer registered at their study practice.

The analyses of referral rates in this paper are restricted to men who were reported to be asymptomatic at the time of their PSA test in each period. The referral rate was defined as the proportion of asymptomatic men with no previous diagnosis of prostate cancer who were referred to urologists by their GPs following a PSA test. The proportions of men referred were studied within groups of men categorised by the age-specific PSA cutoff levels defined by the PCRMP.

In the analyses of data on men's individual records, it was not possible to study variation in referral rates by practice, or between GPs within practices, because of the small number of men being referred. Social deprivation was not included in the analyses, as individual-based data were not collected on social class or postcode, which could have been linked to a social deprivation index.

## RESULTS

### Uptake of practices

Of 71 practices participating in the previous study (Melia *et al*, 2004), 2 no longer existed in the post-launch period, so 69 were invited, of which 48 (70%) took part in the present study. The uptake rate was 75% or more in all areas except Truro, which had an uptake rate of 57% (Fisher's exact test *P*=0.47 comparing the response rate in Truro with the other areas). The invited practices represented 33% of those invited to participate in the previous study. The uptake was low because many non-responding practices were ineligible, that is, did not use the laboratory for all their PSA tests.

The total male populations aged 45–84 years registered at the participating practices were 67 162 and 70 658 men in the pre- and post-launch periods, respectively. There were a total of 200 GP partners at these practices at the time of data collection. The proportions of practices with 1, 2–4, 5–6 and 7–8 or more GP partners were 10% (5), 42% (20), 40% (19) and 8% (4), respectively.

### GP awareness of receiving the PCRMP pack

Awareness of receiving the PCRMP pack was acknowledged by 56% (112 out of 200) of GP partners at the time of data collection with 24 unaware of the pack and 64 who did not know. The proportion of GP partners per practice who were aware of the PCRMP pack varied independently of size of practice. In the five practices with one partner, only one was aware of the PCRMP pack. In the 43 practices with two or more partners, 28 (65%) had ⩾50% of partners aware of the PCRMP pack. Of those with more than one partner, there were 12 (28%) practices where all partners were aware of the pack. In Poisson regression, awareness reported by the GPs did not differ significantly between areas and it was not related to age, gender, MRCGP, number of years working as a GP or number of sessions per week.

### Data completeness

Out of 2494 and 3209 PSA requests in the pre- and post-launch periods, respectively, there were 12 records with missing data on PSA value or patient name, and one record with gender coded female. Record linkage led to 2318 and 3030 men with at least one PSA test in the pre- and post-launch periods, respectively (Table 1). Excluding men reported to be deceased or no longer registered at the practices resulted in pre-launch 515 (36.3%) out of 1420 men with low records and 709 (79.0%) out of 898 men with raised PSA levels available for analysis including those with multiple tests and post-launch 607 (32.4%) out of 1873 and 1040 (89.9%) out of 1157, respectively (Table 1). Data requested on the pro forma on ethnic group and family history of prostate cancer could not be analysed, as high proportions of records had missing data (>30% for ethnicity and 86% for family history).

### Reason for test

The percentage distributions of reason for test were similar in both time periods (Table 2). A higher proportion of men with low PSA levels were asymptomatic compared with men with raised PSA levels (pre-launch: 38 and 19%, respectively, *χ*_1_^2^=56.3, *P*<0.001; post-launch: 39 and 20%, respectively, *χ*_1_^2^=71.2, *P*<0.001).

### Urological referrals in asymptomatic men

The proportions of asymptomatic men referred among those with raised PSA levels did not increase significantly from the pre- to post-launch period (13.7 and 18.2%, respectively, Table 3). There were no referrals in asymptomatic men with low PSA levels. There was also no significant difference in the referral rates by area (Fisher's exact test *P*=0.33). Among those not referred, the most frequent reason was PSA too low (87%, 347 out of 400, post-launch), and re-testing scheduled by GP in general practice (7%, 29 out of 400, post-launch, Table 3). In the raised PSA group, additional reasons for non-referral included serious comorbidity.

Analysis of referral according to the PCRMP age-specific cutoff levels, restricted to men aged 50–84 years, showed that in the pre-launch period no men were referred with PSA levels below the PCRMP cutoff levels, and there was no significant increase in the proportion of men referred over time in this group (0.0–1.6%, Fisher's exact test *P*=0.07). There was also no significant increase in the proportion of men referred among those with PSA levels above the cutoff levels (24.0–29.4%, *P*=0.42, Table 4). No men were referred in the age group 45–49 years below the age range defined by the PCRMP (Table 4). There was also no significant increase in the proportion referred with age (Fisher's exact test *P*>0.20).

Of all 55 men referred, 45 (82%) were referred within 2 months of their test. In pre-launch findings, the median PSA value associated with referral increased with age from 5.2 ng ml^−1^ in 50- to 59-year olds to 6.8 ng ml^−1^ in 70- to 84-year olds. Post-launch findings were similar with the median PSA value associated with referral increasing from 6.2 ng ml^−1^ in 50- to 59-year olds to 13.0 ng ml^−1^ in 70- to 84-year olds. Five men post-launch with PSA levels below the PCRMP age-specific cutoffs were referred: in these men, PSA levels ranged from 3 to 4.9 ng ml^−1^ in the age range 60–84 years).

Among asymptomatic men not referred post-launch but having a PSA level that was above the PCRMP age-specific guidelines, 18 aged 50–59 years had levels ranging from 3 to 8.5 ng ml^−1^, 29 aged 60–69 years had levels ranging from 4 to 9.5 ng ml^−1^ and 30 aged 70–84 years had levels ranging from 5.1 to 23.6 ng ml^−1^. When these data were restricted further to those non-referrals for which the GPs reported the PSA level to be too low for referral, the ranges were 3–5.2, 4–8.9 and 5.3–7.5 ng ml^−1^, respectively.

## DISCUSSION

The aim of this study was to investigate the impact of the PCRMP guidelines for age-specific cutoff levels for PSA levels in asymptomatic men on urological referrals. No significant change in the proportion of asymptomatic men being referred to urologists following a PSA test was observed between the two time periods before and after the launch of the PCRMP. The proportions referred were lower than that anticipated. Thus, the influence of the PCRMP guidelines seems to have been low. The relation of the PCRMP guidelines to decisions about referral could not be studied retrospectively because these data are not routinely recorded. However, at the time of data collection, 56% of GP partners in the study reported being aware that they had received the PCRMP packs, and the proportion of GP partners aware per practice showed a wide variation from 0 to 100%.

GPs adopted a varied but cautious approach to referral, with all non-referral considered by their GP to have a low PSA level having levels below 10 ng ml^−1^. It is not know why some GPs chose to take a PSA test when the patient had comorbidities, which influenced the decision not to refer them. It was not feasible to collect this information, as the GP consulting at the time of the PSA test was not necessarily the one providing the retrospective data. No local guidelines for referral of asymptomatic men were reported. Review of the guidance provided in the study laboratories’ pathology reports was primarily concerned with symptomatic men and those already diagnosed with prostate cancer. Re-testing was the most frequent management in non-referrals in men with raised PSA levels.

The main limitations of the study concern the choice of study design, the uptake of the practices, the completeness of data collection and variation in laboratory assays to measure PSA. It was not possible to conduct a randomised controlled trial because the programme was launched nationwide. The observational design used may have been affected by local and national influences. However, if the PCRMP guidelines had had a strong impact on PSA testing and referrals, this should have been detected.

The data collected on GP partners’ awareness of the PCRMP packs are limited because knowledge and views on the guidelines were not studied in detail. As these data were only available at the time of data collection, it was not appropriate to link them to the retrospective referral data: awareness among GP partners does not imply that the PCRMP packs were used during consultations, and the GPs helping with data collection from patient notes were not always those who had made the referral decisions. It was also not possible to study men's experiences of PSA testing as has been done in one study (Evans *et al*, 2007).

Data collection on referrals was restricted to two identical times of year in 2001/2002 and 2003/2004. This controlled for time of year, and the second period allowed GPs a year after the launch to take on board the PCRMP guidelines. It was not feasible to ask GPs to provide data for a longer time period.

The practices were chosen from our previous study to enable the use of data already collected. The practices in the previous study were not a random sample, representing just more than 30% of those recorded as using the pathology laboratories. The practices in the study of referral rates represent a smaller proportion of single-handed practices (10%) than nationally (27% in 2004) (Department of Health, 2005). They also represented a narrow range of social deprivation scores linked to practice postcodes: the mean (±s.d.) Townsend scores for the participating practices were −2.62±0.08, −1.46±0.99, −1.32±0.50 and −2.16±0.83 in Chichester, Sutton & Merton, Truro and York, respectively. It is possible that the participating GPs were more likely to test for PSA than GPs in more deprived areas, but it is unclear what effect the selection bias could have had on urological referrals and use of the PCRMP guidelines.

Data on referral were incomplete for men who had died or who were no longer registered at the practices, as their notes were not available. The proportions were higher pre-launch than post-launch (13.7%, 194 out of 1418, and 7.9%, 141 out of 1788, respectively, *χ*_1_^2^=28.4, *P*=0.0001). Men with missing data were older and had higher levels of PSA compared with men not missing these data. Thus, those excluded from the analyses were more likely to have been symptomatic or diagnosed with prostate cancer than men for whom data were completed. The results for referral rates in asymptomatic men are less likely to have been affected by these biases than in men tested for other reasons.

All of the laboratories participated in the Quality Assurance scheme for PSA measurement (Milford Ward *et al*, 2001). Their methods of measurement were Bayer Immunol, Bayer Centaur and DPC Immunite (two laboratories). Variation in bias of PSA measurements by these assays (Roddam *et al*, 2006) is likely to have affected a very small proportion of men, as the GPs were not using the same cutoff levels. The reliability of the referral data was strengthened by asking for a date of referral and consultant name. Analysis of reason for test by PSA level showed that low levels were associated with asymptomatic testing, as has been found in screening trials (De Koning *et al*, 2002). The distribution of reason for test in men with low and raised PSA levels was also as expected in both time periods of the study (Table 2).

Few studies have reported on urological referral rates in general practice linked to specific PSA tests, partly because there is considerable workload involved in retrospectively searching patient notes. In the United States (Dyche *et al*, 2006), referral rates were studied from 1998 to 2004 in men aged ⩾75 years with no previous prostate cancer diagnosis attending one centre in Iowa: in those with PSA levels between 0.1 and 4.0 ng ml^−1^, 28.6% were referred, and in those with PSA levels >4.0 ng ml^−1^, 52% were referred. For the comparable populations defined by age and PSA level in our study, 7% (8 out of 120) and 30% (56 out of 186) were referred, respectively, pre-launch, representing the period 2001/2002.

As the rate of PSA testing continues to increase in England, and there is no standard management of tests in asymptomatic men, evaluation of the PCRMP guidelines is essential (Watson *et al*, 2006). Without routine, standardised data on reasons for PSA tests in general practice, it will be impractical to monitor trends in use of the PSA test, and its impact on GP workload and detection of prostate cancer. There is an urgent need for evaluation to ensure future, effective implementation of guidelines.

### Approval

The study was approved by NHS Research Ethics Service (04/MRE01/39) and by the Patient Information Advisory Group (PIAG 2-06(f)/2004).

## Figures and Tables

**Table 1:** Number of men for whom data were collected and counts of exclusions by time period and whether the PSA level was low (<3 ng ml^−1^) or raised (⩾3 ng ml^−1^)

	**Pre-launch, 1 December 2001 to 31 May 2002**			**Post-launch, 1 December 2003 to 31 May 2004**		
	**Total**	**No. with 1st test low record**	**No. with 1st test raised record**	**Total**	**No. with 1st test low record**	**No. with 1st test raised record**
No. of men aged 45–84 years before stratified sampling	2318	1420	898	3030	1873	1157
						
*Reasons for exclusions from laboratory data files*						
Removed by sampling procedure		848	0		1199	0
One practice reduced workload		9	23		11	32
At one lab, GPs only notified of rounded values, so some PSA levels were classified as low		NA	20		NA	0
No. of men aged 45–84 years after stratified sampling	1418	563	855	1788	663	1125
						
*Reasons for exclusion from data analysis after data collection from general practices*						
Men's records not available due to death or no longer registered at practice	194	48	146	141	56	85
*No. of men aged 45–84 years alive and still registered at practice*	1224	515	709	1647	607	1040
Of which, no. with a single test	1111			1520		

GP, general practitioner; PSA, prostate-specific antigen.

**Table 2:** Distribution of men by reason for test, time period and whether they had a low (<3 ng ml^−1^) or raised (⩾3 ng ml^−1^) PSA level

	**Pre-launch, 1 December 2001 to 31 May 2002**				**Post-launch, 1 December 2003 to 31 May 2004**			
	**Low records^a^**		**Raised records**		**Low records^a^**		**Raised records**	
**Reason for test**	**No.**	**%**	**No.**	**%**	**No.**	**%**	**No.**	**%**
Asymptomatic	194	37.7	131	18.5	234	38.6	203	19.5
Symptomatic	188	36.4	284	40.1	201	33.1	445	42.8
Re-test	56	10.9	220	31.0	56	9.2	273	26.3
Prostate cancer already diagnosed	72	14.0	68	9.6	100	16.5	103	9.9
Not known	5	1.0	6	0.8	16	26.4	16	1.5
Total included in data collection	515	100	709	100	607	100	1040	100
Significance of difference in distribution of reason for test between low and raised records	*χ*_4_^2^=100.3, *P*<0.001				*χ*_4_^2^=132.9, *P*<0.001			

PSA, prostate-specific antigen.

No significant differences between the two time periods were found in the distribution of reason for test among men with low or men with raised PSA levels.

a Based on sub-sample, see Table 1.

**Table 3:** Number of urological referral and reasons for non-referral in asymptomatic men by time period and whether they had a low (<3 ng ml^−1^) or raised (⩾3 ng ml^−1^) PSA level

						**Distribution of non-referrals by reason**						
	**Total number of men**	**Referrals**		**Non-referrals**	**Significance of difference in % referred between men with low and raised PSA records**	**PSA too low**	**Re-test scheduled and initiated by**		**Serious comorbidity**	**Other**	**Lost to follow-up**	**Not known**
		**n**	**%**				**GP practice**	**Urologist**				
*Pre-launch*												
Low PSA records	194	0	0	194	*χ*_1_^2^=28.2	187	3	0	0	3	0	1
Raised PSA levels	131	18	13.7	113	*P*<0.001	71	21	2	6	3	3	7
Total	325	18	5.5	307		258	24	2	6	6	3	8
												
Post-launch												
Low PSA records	234	0	0	234	*χ*_1_^2^=46.6	230	3	0	1	0	0	0
Raised PSA levels	203	37	18.2	166	*P*<0.001	117	26	4	6	7	1	5
Total	437	37	8.5	400		347	29	4	7	7	1	5

GP, general practitioner; PSA, prostate-specific antigen.

Significance: no significant differences between the two time periods in distributions of referral and non-referral for either low or raised PSA levels.

**Table 4:** Proportion of asymptomatic men referred pre- and post-launch aged 45–84 years grouped by PSA level and age

**Age (years)**	**45–49**		**50–59**		**60–69**				**70–84**				**Total % men referred below and above age-specific cutoff**	
**PSA level (ng ml^−1^)**	**<3**	**⩾3**	**<3**	**⩾3**	**<3**	**⩾3–<4**	**Total <4**	**Total ⩾4**	**<3**	**⩾3–⩽5**	**Total ⩽5**	**Total >5**	**Below**	**Above**
*Pre-launch*														
%	0	0	0	**37.5**	0	0	0	**22.9**	0	0	0	**16.7**	0	**24**
No. referred	0	0	0	**6**	0	0	0	**8**	0	0	0	**4**	0	**18**
Total no. of men	10	0	65	**16**	61	19	80	**35**	58	37	95	**24**	250	**75**
														
*Post-launch*														
%	0	0	0	**18.2**	0	3.4	0.9	**31**	0	6.5	2.9	**33.3**	1.5	**28.6**
No. referred	0	0	0	**4**	0	1	1	**13**	0	4	4	**15**	5	**32**
Total no. of men	10	3	60	**22**	86	29	115	**42**	78	62	140	**45**	325	**112**

PCRMP, Prostate Cancer Risk Management Programme; PSA, prostate-specific antigen.

Results in bold relate to PCRMP guidelines for referral: aged 50–59 years ⩾3 ng ml^−1^, aged 60–69 years ⩾4 ng ml^−1^ and 70 years or more >5 ng ml^−1^.

No significant difference in the proportion of referrals between pre- and post-launch periods.
